# 2510. Adapting the CDC HCV viral clearance cascade methodology for persons with HIV (PWH) and HCV co-infection in 5 Health Department Jurisdictions in the US

**DOI:** 10.1093/ofid/ofad500.2128

**Published:** 2023-11-27

**Authors:** Maximilian D Wegener, Ralph P Brooks, Lisa G Nichols, Merceditas Villanueva

**Affiliations:** Yale University School of Medicine - AIDS Program, New Haven, Connecticut; Yale School of Medicine - AIDS Program, New Haven, Connecticut; Yale University, New Haven, Connecticut; Yale School of Medicine, New Haven, Connecticut

## Abstract

**Background:**

Cascades of care have been used by health departments to evaluate outcomes and gaps in HIV care and until recently, no standard existed for HCV cascades. The CDC guidance for HCV viral clearance cascades allows standardized use of HCV surveillance data. We adapted this approach for persons with HIV/HCV coinfection for 5 health jurisdictions in the US.

**Methods:**

Lists of coinfected persons were generated by matching HCV surveillance data to prevalent HIV surveillance (eHARS) as of 12/31/2019. Dispositions based on HCV test results were created for viral clearance cascade steps: ever infected, viral testing, initial infection, cleared, and persistent infection. We collected aggregate data from each jurisdiction’s prevalent HIV, HCV, and coinfected populations. Quarterly updated data were submitted and analyzed from 12/31/2019-12/31/2021.

**Results:**

Overall, jurisdictional coinfected populations were White (61%), Male [sex at birth] (75%), over 45 (76%) with IDU as HIV transmission risk factor (44%). As of most recent submission: virally tested was 73% (jurisdictional range 65-88%) of ever-infected; initial infection 83% (81-89%) of virally tested; cleared 44% (21-63%) of initial infection. Those more likely to receive viral testing include persons over 45, IDU, and those with more recent HCV and HIV lab tests; less likely were MSM (HIV transmission) along with Asian and Native American persons. Those more likely to achieve HCV clearance include those over 45, those with more recent HCV and HIV lab tests, and persons with undetectable HIV viral loads; less likely were Transgender persons.

**Conclusion:**

Diverse health jurisdictions successfully developed data strategies including enhancing HCV surveillance databases to implement the CDC HCV viral clearance cascade adapted for persons with HIV/HCV co-infection. Jurisdictions are now able to not only assess HCV cure rates of their co-infected population but testing and treatment gaps as well. The viral clearance cascades can provide key implementation strategy data for HCV microelimination through the development of innovative engagement strategies focused on the identified gaps.

**Disclosures:**

**Ralph P. Brooks, MS**, Merck: Stocks/Bonds

